# Molecular determinants of Ca^2+^ sensitivity at the intersubunit interface of the BK channel gating ring

**DOI:** 10.1038/s41598-017-19029-8

**Published:** 2018-01-11

**Authors:** Qin Li, Yingxin Li, Hua Wei, Hao-Min Pan, Alexandre G. Vouga, Brad S. Rothberg, Yunkun Wu, Jiusheng Yan

**Affiliations:** 1 Department of Anesthesiology and Perioperative Medicine, The University of Texas MD Anderson Cancer Center, Houston, Texas USA; 2 Department of Medical Genetics and Molecular Biochemistry, Temple University Lewis Katz School of Medicine, Philadelphia, Pennsylvania USA; 3 State Key Laboratory of Structural Chemistry, Fujian Institute of Research on the Structure of Matter, Chinese Academy of Sciences, Fuzhou, China; 4 Present Address: Cardiovascular Institute, Stanford University School of Medicine, Stanford, California, USA; 5 Present Address: University of Pennsylvania Health System, Philadelphia, Pennsylvania USA

## Abstract

The large-conductance calcium-activated K^+^ (BK) channel contains two intracellular tandem Ca^2+^-sensing RCK domains (RCK1 and RCK2), which tetramerize into a Ca^2+^ gating ring that regulates channel opening by conformational expansion in response to Ca^2+^ binding. Interestingly, the gating ring’s intersubunit assembly interface harbors the RCK2 Ca^2+^-binding site, known as the Ca^2+^ bowl. The gating ring’s assembly interface is made in part by intersubunit coordination of a Ca^2+^ ion between the Ca^2+^ bowl and an RCK1 Asn residue, N449, and by apparent intersubunit electrostatic interactions between E955 in RCK2 and R786 and R790 in the RCK2 of the adjacent subunit. To understand the role of the intersubunit assembly interface in Ca^2+^ gating, we performed mutational analyses of these putative interacting residues in human BK channels. We found that N449, despite its role in Ca^2+^ coordination, does not set the channel’s Ca^2+^ sensitivity, whereas E955 is a determinant of Ca^2+^ sensitivity, likely through intersubunit electrostatic interactions. Our findings provide evidence that the intersubunit assembly interface contains molecular determinants of Ca^2+^-sensitivity in BK channels.

## Introduction

Large-conductance, Ca^2+^-activated K^+^(BK) channels (also called Maxi-K, Slo1, and KCa1.1 channels), which are characterized by large, single-channel conductance and dual activation by membrane voltage and intracellular Ca^2+^ ^1–4^, are critically involved in diverse physiological processes. In the brain, BK channels regulate neuronal firing and neurotransmitter release^5–7^ and are involved in motor coordination^8^, rhythmic control of the circadian clock^9^, as well as frequency tuning of cochlear hair cells^10^. Mutations or dysregulation of neuronal BK channels can cause epilepsy and paroxysmal dyskinesia^11,12^. BK channels control the contractile tone of smooth muscle and are involved in the regulation of blood pressure^13^, bladder contractility^14^, and erectile function^15^. In non-excitable secretory epithelial cells, BK channels provide an essential pathway for resting K^+^ efflux^16^.

BK channels are composed of homotetramers of the pore-forming, Ca^2+^- and voltage-sensing α subunits either alone or with regulatory β or γ subunits^17–19^. Each α subunit contains a transmembrane domain for voltage sensing and channel pore formation and a large cytosolic C-terminus composed of two RCK domains (RCK1 and RCK2) for Ca^2+^ and Mg^2+^ sensing^3,20–26^. BK channels possess many biophysical features that make them an ideal system for studying ion channel gating mechanisms^27^. The biophysical mechanism of BK channel activation by voltage and Ca^2+^ can be described by a well-established allosteric gating model, in which the channel pore opening is allosterically regulated by the movement of each voltage sensor and the binding of Ca^2+^ at each Ca^2+^ sensor on the four subunits in a largely independent manner^27,28^. X-ray crystallography of BKα C-termini in humans and zebrafish^20,21^ and cryo-electron microscopy (cryo-EM) of the entire BKα channel in *Aplysia californica*^22,23^ at near-atomic resolutions have revealed that the tandem RCK1 and RCK2 domains from four individual subunits are organized into a gating ring of eight RCK domains by tetramerization at the intersubunit assembly interface. In each subunit, the two RCK domains are assembled into a pseudo-dimeric bi-lobed architecture^21^ harboring a Ca^2+^-binding site in the RCK1 domain and another Ca^2+^-binding site, known as the Ca^2+^ bowl, formed by a string of Asp residues in the RCK2 domain^29,30^. Comparisons of the BK channel structures in the absence and presence of Ca^2+^ indicate that the gating ring expands in response to Ca^2+^ binding and propagates conformational changes to the channel pore^20,22^, suggesting a likely involvement of the interfaces between the RCK domains of adjacent subunits, known as the “intersubunit assembly interface”^20,21^, in BK channel activation by Ca^2+^.

To probe the role of the intersubunit assembly interface in BK channel Ca^2+^ gating, we perturbed the structure of the interface by eliminating intersubunit Ca^2+^ coordination mediated by N449 residues, and by disrupting intersubunit electrostatic interactions mediated by E955 residues, in human BK channels. We found that the Ca^2+^-coordinating N449 residue has little role in determining BK channel Ca^2+^ sensitivity. However, E955 plays an important role in determining BK channel Ca^2+^-sensing likely through intersubunit electrostatic interactions, by a mechanism that is mediated in part through the Ca^2+^ bowl.

## Results

### Intersubunit interactions at the BK gating ring’s assembly interface

**Figure 1:**
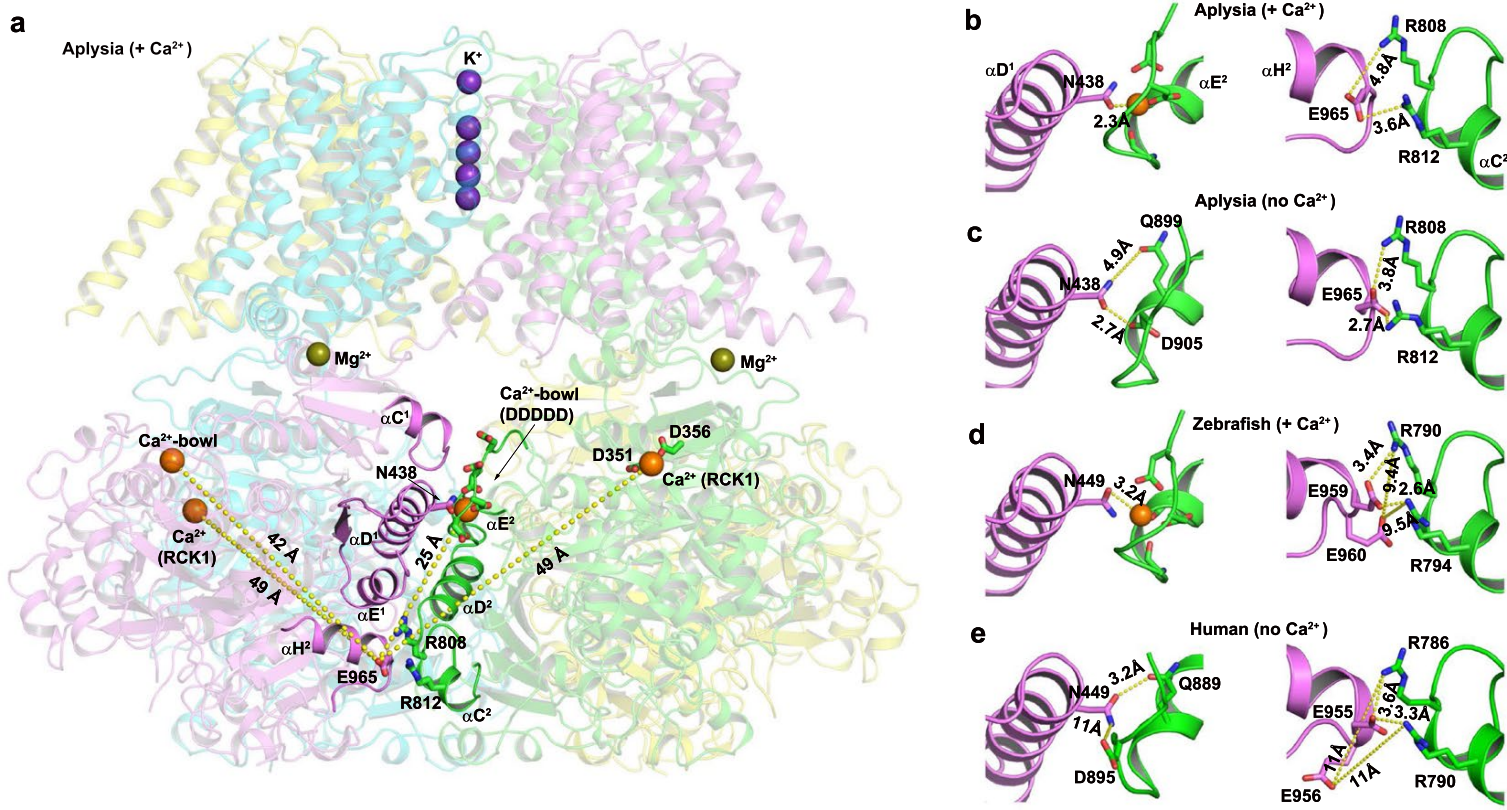
Structures of the BK channel gating ring’s intersubunit assembly interface. (**a**) Structures of an intersubunit assembly interface (shown in normal cartoon mode) in the context of the whole *Aplysia californica* BK channel (depicted in transparent cartoon mode). For clarity, Ca^2+^ and Mg^2+^ ions are shown only in the two front subunits, and the residues relevant to this study are shown in stick mode. (**b–e**) Local structures of the intersubunit interactions between the Ca^2+^ bowl site and the hN449 residue or its equivalent residues (left) and intersubunit electrostatic interactions of hE955-hR786/hR790 residues or their equivalent residues (right) of BK channels in *Aplysia californica* with Ca^2+^ (**b**) and without Ca^2+^ (**c**); in zebrafish with Ca^2+^ (**d**); and in humans without Ca^2+^ (**e**). For the Ca^2+^ bowl site with Ca^2+^, the side-chains or main-chains of the Ca^2+^-coordinating residues are also shown in stick mode. The structural cartoons were drawn with pyMOL using cryo-EM BK channel structures of *Aplysia californica* (PDB IDs: 5TJ6 and 5TJI)^22,23^ and X-ray crystal structures of the BK channel gating rings of zebrafish (PDB ID: 3U6N)^20^ and humans (PDB ID: 3NAF)^21^.

We first examined the structural features of the gating ring’s intersubunit assembly interface. Our examination considered all reported near-atomic-resolution structural data of BK channel gating rings, including X-ray crystal structures of the human BK channel Ca^2+^-free gating ring^21^ and the zebrafish Ca^2+^-bound gating ring^20^, and cryo-EM structures of the full-length *Aplysia californica* BK channel in the presence and absence of Ca^2+^ ^22,23^. The intersubunit assembly interface is formed by the RCK1 domain’s α-helices C, D, and E (αC^1^, αD^1^ and αE^1^) and the RCK2 domain’s α-helix H (αH^2^) in one subunit and the RCK2 domain’s Ca^2+^ bowl and α-helices C, D, and E (αC^2^, αD^2^, and αE^2^) in the neighboring subunit (Fig. 1a). Although the hydrophobic interactions among αD^1^, αE^1^, αD^2^, and αE^2^ dominate at the core of the intersubunit assembly interface^21^, two types of non-hydrophobic interactions may contribute to the stability and/or function of the gating ring’s tetrameric structure. On the more upper side of the intersubunit assembly interface, where the Ca^2+^ bowl site is situated, an Asn residue (N449 in humans and zebrafish and N438 in *Aplysia*) in the αD^1^ from one subunit provides additional coordination for the Ca^2+^ atom of the neighboring subunit’s Ca^2+^ bowl (Fig. 1a,b and d). In the absence of Ca^2+^, this Asn residue is close to residues of the Ca^2+^ bowl site (D905 in *Aplysia* and Q889 in humans) (Fig. 1c and d). On the lower side of the intersubunit assembly interface, electrostatic interactions are formed by a negatively charged Glu residue (E955 in humans, E965 in *Aplysia*, and E959 in zebrafish) located on αH^2^ of one subunit (on the same side as the human N449 residue) and two positively charged Arg residues (R786 and R790 in humans, R808 and R812 in *Aplysia*, and R790 and R794 in zebrafish) located on αC^2^ of the neighboring subunit (on the same side as the Ca^2+^ bowl) (Fig. 1). In all reported structures, in the absence and presence of Ca^2+^, the Glu residue (E955 in humans) is close (within 3.4 Å) to the two Arg residues (R786 and R790 in humans) to allow these residues form intersubunit electrostatic interactions (Fig. 1b–e). The above-mentioned four residues (N449, E955, R786, and R790 in humans) are fully conserved across different species. The BK channels of most species, including humans and zebrafish, have an additional negatively charged Glu residue (E956 in humans and E960 in zebrafish; substituted by G966 in *Aplysia*), which is immediately next to the other Glu residue (E955 in humans, E959 in zebrafish) in the amino acid sequence but with its side chain positioned approximately 10 Å from the other subunit’s two Arg residues (R786 and R790 in humans) (Fig. 1a,d and e). These intersubunit non-hydrophobic interacting residues likely play a role in stabilizing the intersubunit assembly of the gating ring, and the intersubunit Ca^2+^-coordinating Asn residue may also directly affect Ca^2+^ affinity at the Ca^2+^ bowl site. Structural perturbation by mutations at these residues may be helpful in understanding the role of the gating ring’s intersubunit assembly interface in BK channel gating by Ca^2+^.

### Neutralization of E955 increases the intrinsic channel-pore opening but greatly decreases Ca^2+^ sensitivity in human BK channels

**Figure 2:**
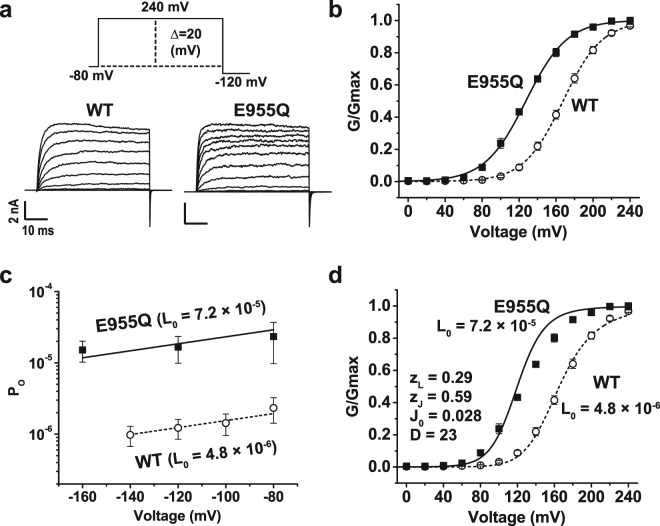
Effects of E955Q on the voltage-dependence of BK channel activation in the absence of Ca^2+^. (**a**) The voltage protocol used for patch-clamp recording and the obtained representative currents of the E955Q and WT channels. (**b**) Voltage dependence of BK channel activation fitted with a single Boltzmann function. (**c**) The channel’s P_O_ measured at very negative voltages fitted with the HA model. (**d**) Comparison of the experimental data (scattered data) and the HA model simulated data (lines) using the L_0_ values shown in (**c**). The gating parameter values used for z_L_ and z_J_ were from the previously established^27^ and J_0_ and D were obtained by a best fit of the WT data.

To determine the role of the negative charge of E955 on human BK channel gating, we neutralized it to Gln and found that E955Q shifted the conductance-voltage (G-V) curve towards the hyperpolarizing direction by 40 mV in the absence of Ca^2+^ (V_1/2_ = 167.5 ± 2.4 mV for wild type [WT] vs 127.2 ± 1.4 mV for E955Q) (Fig. 2a and b; Supplementary Table S1 and Fig. S1). Because the intracellular RCK domains are immediately connected to the channel’s pore through an S6-RCK1 linker region, E955Q may affect the channel pore’s intrinsic equilibrium constant (L) for the closed-open (C-O) transition. In the context of the Horrigan-Aldrich (HA) model of BK channel gating^27^, a change in L is proportional to the change in the channel’s open probability (P_O_) in the absence of Ca^2+^ at very negative voltages where the voltage sensors are forced into resting states. The relationship between P_O_ and L can be described by the equation P_O_ = L = L_0_exp(z_L_V/kT), in which L_0_ is L at 0 voltage and z_L_ is the partial charge associated with channel opening. To examine a possible effect of E955Q on L, we measured the channel’s P_O_ at very negative voltages (e.g., −160 mV to −80 mV) and found that it was increased by approximately 10-fold in E955Q channels compared with WT channels (Fig. 2c). By fitting the data using the previously established z_L_ value (z_L_ = 0.29 *e*)^27^, we estimated that L_0_ is increased from 4.8 × 10^−6^ in WT to 7.2 × 10^−5^ in E955Q channels (Fig. 2c). Assuming that all other gating parameters are not changed, such a change in L_0_ can largely explain the E955Q-induced, approximately 40-mV shift in V_1/2_ towards the hyperpolarizing direction in the absence of Ca^2+^ (Fig. 2d).

**Figure 3:**
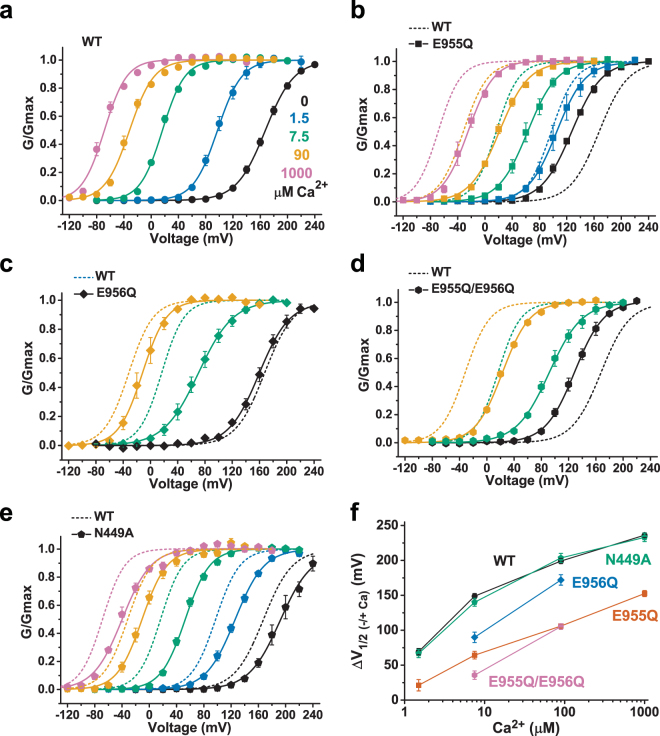
Effects of E955Q, E9556Q, and N449A mutations on the Ca^2+^ sensitivity of BK channels. (**a–e)**, Voltage dependence of BK channel activation for the WT (**a**) and E955Q (**b**), E956Q (**c**), E955Q/E956Q (**d**), and N449A (**e**) mutant channels at different Ca^2+^ concentrations. (**f)** Relationships between the ΔV_1/2 (−/+Ca)_ and Ca^2+^ for WT and mutant channels. For comparison, the voltage dependence of the WT channel is shown as dashed fitted lines in panels (b–e).

Over Ca^2+^ concentrations ranging from 1.5 µM to 1 mM, we observed that the E955Q mutation greatly decreased the apparent Ca^2+^-sensitivity of BK channels. In the WT channels, the Ca^2+^-induced shifts in V_1/2_ (ΔV_1/2 (−/+Ca)_ = V_1/2(0 Ca)_ − V_1/2 (+ Ca)_) were 69 mV at 1.5 μM Ca^2+^ (V_1/2_ = 98.2 ± 4.0 mV), 149 mV at 7.5 µM Ca^2+^ (V_1/2_ = 18.7 ± 2.8 mV), 199 mV at 90 μM Ca^2+^ (V_1/2_ = −31.6 ± 2.8 mV), and 236 mV at 1 mM Ca^2+^ (V_1/2_ = −68.7 ± 2.2 mV) (Fig. 3a). However, the ΔV_1/2 (−/+Ca)_ values of E955Q mutant channels were reduced to 21 mV at 1.5 μM Ca^2+^ (V_1/2_ = 106.0 ± 8.0 mV), 64 mV at 7.5 µM Ca^2+^ (V_1/2_ = 62.9 ± 5.2 mV), 106 mV at 90 μM Ca^2+^ (V_1/2_ = 21.3 ± 2.6 mV), and 153 mV at 1 mM Ca^2+^ (V_1/2_ = −25.3 ± 4.1 mV), which were decreased by 70% (48 mV), 57% (85 mV), 47% (93 mV), and 35% (84 mV), respectively, compared with those of WT channels (Fig. 3b and f). To determine whether the negative charge of the immediately neighboring E956 residue can also affect Ca^2+^ sensing, we mutated E956 to Gln. In the absence of Ca^2+^, the E956Q mutant channel had a V_1/2_ value of 160.4 ± 4.2 mV, which was close to that of the WT channel (Fig. 3c). In the absence and presence of Ca^2+^, the ΔV_1/2 (−/+Ca)_ values of the E956Q channel were 90 mV at 7.5 μM Ca^2+^ (V_1/2_ = 70.6 ± 6.3 mV) and 172 mV at 90 μM (V_1/2_ = −11.6 ± 6.2 mV), which were decreased by 40% (59 mV) and 13% (27 mV), respectively, compared with those of the WT channel (Fig. 3c and f). The combination of these two mutations (E955Q/E956Q) abolished most of the channel-activating effect of 7.5 μM Ca^2+^ (V_1/2_ = 92.8 ± 4.6 mV) with a resultant ΔV_1/2 (−/+Ca)_ value of 36 mV, which was 76% (113 mV) less than that of the WT channels and 45% (29 mV) less than that of the E955Q channels (Fig. 3d and f). At 90 μM Ca^2+^, the E955Q/E956Q channel had a V_1/2_ value of 23.2 ± 1.1 mV and thus a ΔV_1/2 (−/+Ca)_ value of 105 mV, which was similar to that of the E955Q channel (Fig. 3d and f). These results suggest that E956Q also reduced the BK channel Ca^2+^ sensitivity, albeit to a much smaller extent than E955Q did, and that these two mutations’ effects on BK channel Ca^2+^gating are largely independent and additive at the tested two Ca^2+^ concentrations.

### The Ca^2+^-coordinating N449 residue is not a determinant of Ca^2+^ sensitivity

Although E955Q greatly impairs the human BK channel’s Ca^2+^ sensing, the equivalent residue (e.g., E965 in *Aplysia* BK channels) is located more than 20 Å from the nearest Ca^2+^ in the Ca^2+^ bowl site in the gating ring structures of *Aplysia* (Fig. 1a) and zebrafish. To determine whether the N449 residue, whose equivalents in *Aplysia* (N438) and zebrafish (N449) were found to directly coordinate Ca^2+^ in the Ca^2+^ bowl (Fig. 1b and d), may affect human BK channel Ca^2+^ gating, we eliminated the residue’s Ca^2+^-coordinating capacity by a substitution with Ala. We found that, compared with the G-V curve of WT channels (V_1/2_ = 167.5 ± 2.4 mV), the G-V curve of the N449A channels was shifted to the depolarizing direction by approximately 30 mV (V_1/2_ = 193.2 ± 5.3 mV) in the absence of Ca^2+^ (Fig. 3e). However, the Ca^2+^ sensitivity remained largely unchanged in the N449A mutant channel, whose G-V curves were shifted to the hyperpolarizing direction by Ca^2+^ to an extent similar to that observed in the WT channels (Fig. 3e). The observed ΔV_1/2 (−/+Ca)_ values of the N449A mutant channels were 67 mV at 1.5 µM Ca^2+^ (V_1/2_ = 126.5 ± 2.6 mV), 140 mV at 7.5 µM Ca^2+^ (V_1/2_ = 53.1 ± 2.0 mV), 204 mV at 90 µM Ca^2+^ (V_1/2_ = −10.3 ± 3.9 mV), and 233 mV at 1 mM Ca^2+^ (V_1/2_ = −39.8 ± 2.9 mV), all of which were similar to those of the WT channels (Fig. 3f and Fig. S1). These data suggest that N449 plays little role in BK channel Ca^2+^ binding despite the residue’s observed Ca^2+^ coordination at the Ca^2+^ bowl site in the reported structures of BK channels in both *Aplysia* and zebrafish. Thus, N449’s impact on Ca^2+^sensitivity is in stark contrast to that of the distally localized E955 and E956.

### E955Q affects Ca^2+^ sensing at the Ca^2+^ bowl site

**Figure 4:**
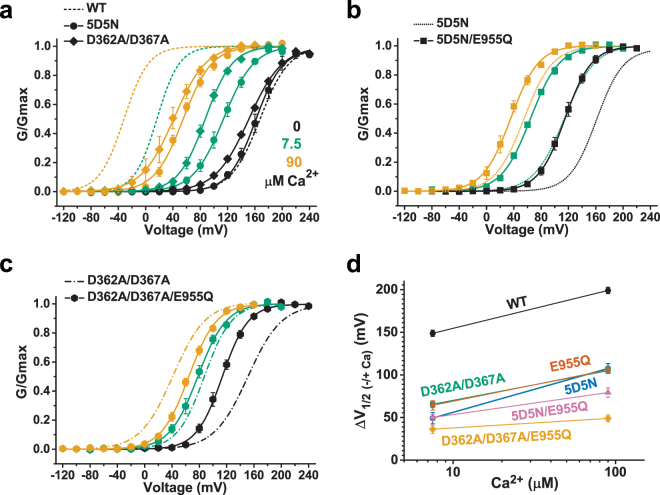
Effects of E955Q on the Ca^2+^ sensitivity of BK channels without a functional RCK1-Ca^2+^ site or Ca^2+^ bowl site. (**a**–**c)** Voltage dependence of BK channel activation at 0, 7.5, and 90 µM Ca^2+^ for the RCK1-Ca^2+^ site–deficient D362A/D367A mutant and the Ca^2+^ bowl site–deficient 5D5N mutant (**a**), the 5D5N/E955Q mutant (**b**), and the D362A/D367A/E955Q mutant (**c**). For comparison, the voltage dependence of the reference channels (WT or mutant) is shown as dashed fitted lines. (**d)** Relationships between the ΔV_1/2 (−/+Ca)_ and Ca^2+^ for WT and mutant channels.

To determine whether the E955Q mutation affects Ca^2+^ sensing preferentially through the RCK1 site or the Ca^2+^ bowl, we assessed its effects on Ca^2+^ sensing–deficient channels that lack functional Ca^2+^ bowl sites owing to the D894N/D895N/D896N/D897N/D898N (5D5N) mutation or that lack RCK1 Ca^2+^ sites owing to the D362A/D367A mutation. Similar to those reported previously^25,30,31^, the 5D5N and D362A/D367A mutant channels were much less responsive to Ca^2+^ than the WT channels, as they encountered decreases in ΔV_1/2 (−/+Ca)_ at 7.5 µM Ca^2+^ (ΔV_1/2 (−/+Ca)_ = 49 and 66 mV) of 67% (100 mV) and 56% (83 mV), respectively, and at 90 µM Ca^2+^ (ΔV_1/2 (−/+Ca)_ = 108 and 105 mV) of 46% (92 mV) and 47% (94 mV), respectively (Fig. 4a and d). The E955Q mutant channel was thus similar or comparable to each of these Ca^2+^ binding–defective mutant channels in terms of their decreased Ca^2+^ sensitivity (Fig. 4d). We found that the 5D5N/E955Q channel was similar to the 5D5N channel in the Ca^2+^-induced shift in G-V curves at 7.5 µM Ca^2+^ and became moderately less sensitive to 90 µM Ca^2+^ than the 5D5N channel, as evidenced by a decrease of 26% (28 mV) in ΔV_1/2 (−/+Ca)_ (Fig. 4b and d; Fig. S1). However, the D362A/D367A/E955Q mutant channel had greatly reduced Ca^2+^ sensitivity compared with the D362A/D367A channel, as evidenced by a decrease of 45% (29 mV) in ΔV_1/2 (−/+Ca)_ at 7.5 µM Ca^2+^ and a decrease of 53% (56 mV) in ΔV_1/2 (−/+Ca)_ at 90 µM Ca^2+^ (Fig. 4c and d; Fig. S1). These results are consistent with the idea that the effects of E955Q may arise mostly from an influence on the Ca^2+^ bowl site, but are largely independent of the RCK1 site.

### E955Q may affect BK channel gating by disrupting the gating ring’s intersubunit electrostatic interactions

**Figure 5:**
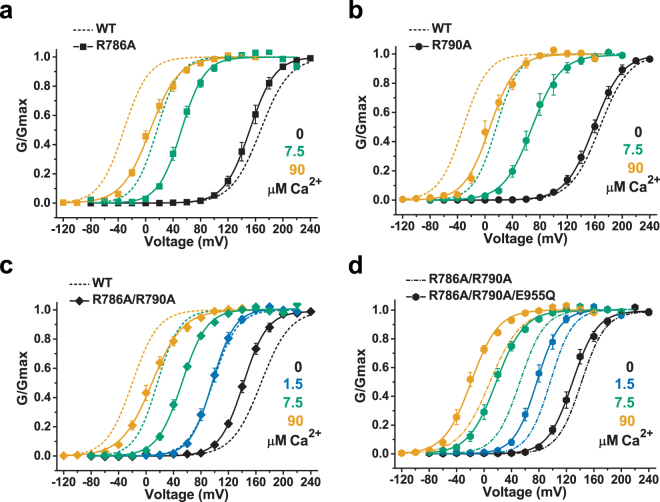
Effects of R786A and R790A mutations on the Ca^2+^ sensitivity of WT and E955Q mutant channels. (**a**–**d)**, Voltage dependence of BK channel activation at 0, 1.5, 7.5, and 90 µM Ca^2+^ for R786A (**a**), R790A (**b**), R786A/R790A (**c**), and R786A/R790A/E955Q (**d**) mutant channels. For comparison, the voltage dependence of the reference channels (WT or mutant) is shown as dashed fitted lines.

To evaluate whether E955Q affects human BK channel gating by disrupting intersubunit electrostatic interactions, we simply neutralized its electrostatic interacting partners, R786 and R790, by mutation to Ala because of the lack of a suitable amino acid substitution (e.g., E → Q) that could neutralize the charge but cause no other changes in the structure or physical and chemical properties of the side-chain. Compared with that of the WT channels, the ΔV_1/2 (−/+Ca)_ values of channels with the single R786A or R790A mutations or the double R786A/R790A mutation were decreased by 25% (37 mV), 40% (60 mV), and 39% (59 mV), respectively, at 10 µM Ca^2+^ and by 27% (54 mV), 24% (48 mV), and 33% (67 mV), respectively, at 90 µM Ca^2+^ (Fig. 5a–c). Thus, similar to the E955Q mutation, the substitution of R786 and R790 with Ala also decreased the apparent Ca^2+^ sensitivity of BK channels. Importantly, in the presence of the R786A/R790A double mutation, the E955Q mutation lost its capability to reduce the BK channel’s apparent Ca^2+^ sensitivity. The observed effects of different concentrations of Ca^2+^ on BK channel activation for the R786A/R790A/E955Q triple mutant channel (ΔV_1/2 (−/+Ca)_ = 52 mV, 112 mV, and 148 mV at 1.5, 7.5, and 90 µM Ca^2+^, respectively) were either similar to (i.e., within 10 mV) or slightly larger than (i.e., within 10 to 25 mV) those of the R786A/R790A double mutant channel (ΔV_1/2 (−/+Ca)_ = 46 mV, 90 mV, and 133 mV at 1.5, 7.5, and 90 µM Ca^2+^, respectively) (Fig. 5d; Fig. S1). The E955Q-induced shift in V_1/2_ in the absence of Ca^2+^ also was largely diminished in the presence of the R786A/R790A mutation (ΔV_1/2 (−/+E955Q)_ = 13 mV) (Fig. 5d). These results are consistent with the notion that E955Q exerts its influence on BK channel voltage and Ca^2+^ gating by disrupting the intersubunit electrostatic interactions of E955 with R786 and R790. That the R786A/R790A double mutation (Fig. 5c) had a smaller effect on BK channel Ca^2+^ gating than the E955Q mutation did (Fig. 3b) could have been due to complications from the mutation-induced structural changes of these two residues that are not limited to charge neutralization.

## Discussion

In structures of Ca^2+^-binding molecules and proteins^28,32^, Ca^2+^ ions are preferentially coordinated by six to eight oxygen atoms. In the BK channel structures of both *Aplysia* and zebrafish^20,22^, the Ca^2+^ ion of the Ca^2+^ bowl is coordinated by seven oxygen atoms: one from the side-chain carboxylate of N449, four from the side-chain carboxylates of D895 and D897, and two from the backbone carbonyl oxygen atoms of Q889 and D892 (Fig. 1b and d). Among these residues, N449 is unique in that it functions as both a Ca^2+^-coordinating residue and an intersubunit bridging residue. Among the three residues coordinating Ca^2+^ with their side-chains, both D895 and D897 are essential to the function of the Ca^2+^ bowl^29^; a functional analysis of N449 has not been reported. N449 is located on the RCK1 N-lobe. Based on the cryo-EM structure of the *Aplysia* BK channel in the presence of Ca^2+^, it was proposed that the RCK1 N-lobe moves to its open conformation as N438 (equivalent to N449 in humans) approaches and coordinates the Ca^2+^ ion in the neighboring subunit’s Ca^2+^ bowl site^23^. Interestingly, we found that N449 does not contribute overall to the BK channel’s Ca^2+^ sensitivity, as N449A mutant channels and WT channels had similar ΔV_1/2 (−/+Ca)_ values over a wide range of Ca^2+^ concentrations (1.5 µM to 1 mM). This finding argues against an obligatory functional role of N449 residues in Ca^2+^-binding affinity^23^. Consistent with our finding, in Ca^2+^-free structures (Fig. 1c and e), this Asn residue still closely interacts with the Ca^2+^ bowl site via Q889 (3.2 Å) in human BK channels^21^ and D905 (2.3 Å) in *Aplysia* BK channels^22^. The ineffectiveness of the N449A mutation in altering Ca^2+^-sensitivity may therefore arise from an energetically greater contribution of other Ca^2+^-coordinating interactions and/or other intersubunit interactions at the interface that are stabilized in the Ca^2+^ bound state.

In contrast, the E955Q mutation, located at the intersubunit assembly interface at a site that is distal (>20 Å) to the Ca^2+^ ion, caused a decrease in the Ca^2+^ sensitivity, to an extent similar to that of the two RCK1-Ca^2+^ and Ca^2+^ bowl null mutants (D362A/D367A and 5D5N) (Figs 3 and 4). We found that E955Q affected the Ca^2+^ sensing of the Ca^2+^ bowl site more than it did that of the RCK1-Ca^2+^ site (Fig. 4). In the *Aplysia* BK channel structure, the equivalent residue (E965) of human E955 is more than 40 Å from the RCK1-Ca^2+^ site of both the same and the neighboring subunits (Fig. 1a). Although the Ca^2+^ bowl is also more than 20 Å from the E955 residue, both E955 and the Ca^2+^ bowl reside at the gating ring’s intersubunit assembly interface. In previously reported structures, E955 or its equivalent residue is positioned within 3.4 Å of R786 and R790 or their equivalent residues and thus may form electrostatic interactions with them (Fig. 1b–e)^20–23^. Thus, it is reasonable to hypothesize that E955 exerts its influence on Ca^2+^ sensitivity via intersubunit electrostatic interactions. Our data are consistent with this possibility, as neutralization of the putative electrostatic interacting partners R786 and R790, diminishes the effect of the E955Q mutation on Ca^2+^ sensitivity (Fig. 5).

How the intersubunit E955-R786/R790 electrostatic interactions at the intersubunit assembly interface might affect Ca^2+^ sensitivity and the function of the Ca^2+^ bowl remains unclear. Because the distances between E955 and R786 and R790 appear similar in the absence or presence of Ca^2+^ in reported structures (Fig. 1b–e), it is not clear that dynamic changes in these interactions are involved in modulating Ca^2+^ gating. Given that extensive intersubunit hydrophobic interactions exist in the core of the assemble interface, disruption of the E955-R786/R790 electrostatic interactions would not be expected to cause disassembly of the gating ring in an intact channel. The E955-R786/R790 electrostatic interactions appear to govern the intersubunit interactions between αH2 and αC2 at the bottom and peripheral edge of the gating ring. The E955Q mutation could result in separation of αH2 and αC2 from the assembly interface and thus likely impair BK channel Ca^2+^ gating via a more local effect on Ca^2+^-bowl function and/or a global influence on the allosteric coupling of Ca^2+^-binding to pore-opening. E955Q also promotes channel opening in the absence of Ca^2+^. Given that Ca^2+^ binding can propagate the conformational change from the local Ca^2+^-binding sites to the distant channel pore to promote the latter’s opening, it is not surprising that structural perturbation of the intracellular domain by mutations outside of the Ca^2+^-binding sites can also affect the channel pore opening.

It is worth noting that the effects of the R786A/R790A double mutation on Ca^2+^-sensing were smaller than those of E955Q, suggesting that disruption of electrostatic interactions may not solely explain the double mutation’s effects. Other R786A/R790A –induced structural changes, e.g., alterations in the side chain size and hydrophobicity, at these two residue sites likely also exert an impact on BK channel Ca^2+^-sensing. It was previously reported that intra-subunit hydrophobic interactions between two RCK domains were critical for Ca^2+^-sensing in BK channels^33^. Although the detailed mechanism remains unknown for the observed effects of mutations at these charged residue sites, our results suggest that the intersubunit assembly interface plays an important role in Ca^2+^ sensing, particularly at the Ca^2+^ bowl site in BK channels, likely by stabilizing the structure at the intersubunit assembly interface.

## Materials and Methods

### Heterologous expression of BK channels in culture cells

Expression constructs of BK channel mutants were made with the QuickChange site-directed mutagenesis kit (Stratagene) using the recombinant cDNA plasmid HF1-hSlo1, which encodes the c-Myc-tagged human BK channel α subunit (GenBank accession number AAB65837), as a template as describe previously^19^. WT and mutant BK channels were heterologously expressed in HEK-293 cells (ATCC). Cells were transfected with the designed plasmid(s) using Lipofectamine 2000 (Invitrogen) and used in electrophysiological assays within 16–72 hours.

### Electrophysiology

BK channel K^+^ currents were measured by patch-clamp recording in excised inside-out patches of HEK-293 cells with symmetric K^+^ solutions of 136 mM KMeSO_3_, 6 mM KCl, and 20 mM HEPES (pH 7.20). Data were acquired at room temperature using an EPC-10 patch clamp amplifier (HEKA). To obtain the desired concentration of free Ca^2+^, the internal solution was supplemented with a certain amount of CaCl_2_ buffered by 5 mM HEDTA or nitrilotriacetic acid. No chelator was used for 1 mM internal Ca^2+^. The free Ca^2+^ concentration was measured with a Ca^2+^-sensitive electrode (Orion Research Inc.). The steady state of channel activation was expressed as G/Gmax, calculated from the relative amplitude of the tail currents (deactivation, held at −120 or −150 mV). The voltage of half maximal activation (V_1/2_) and the equivalent gating charge (z) were obtained by fitting the relationship of G/Gmax with voltage with a single Boltzmann function: G/Gmax = 1/(1 + exp-zF(V − V_1/2_)/RT).

### Data availability

All data generated or analyzed during this study are included in this published article and its Supplementary Information.

## Electronic supplementary material

Supplementary Information
