# Supplementary material for: Molecular determinants of Ca2+ sensitivity at the intersubunit interface of the BK channel gating ring
Source: Sci Rep. 2018 Jan 11;8:509. doi: 10.1038/s41598-017-19029-8 (PMC5765161; doi:10.1038/s41598-017-19029-8)
Supplement: Supplementary file 1 — Supplementary Information [file 41598_2017_19029_MOESM1_ESM.pdf]

Molecular determinants of Ca^{2+} sensitivity at the intersubunit interface of the BK channel gating ring

Qin Li[1], Yingxin Li[1,4] , Hua Wei[1], Hao-Min Pan[1,5] , Alexandre G. Vouga[2], Brad S. Rothberg[2], Yunkun Wu[3], and Jiusheng Yan[1*]

[1]Department of Anesthesiology and Perioperative Medicine, The University of Texas MD Anderson Cancer Center, Houston, Texas, USA.

[2]Department of Medical Genetics and Molecular Biochemistry, Temple University Lewis Katz School of Medicine, Philadelphia, Pennsylvania, USA.

[3]State Key Laboratory of Structural Chemistry, Fujian Institute of Research on the Structure of Matter, Chinese Academy of Sciences, Fuzhou, China.

[4]Current affiliation: Cardiovascular Institute, Stanford University School of Medicine, Stanford, California, USA.

[5]Current affiliation: University of Pennsylvania Health System, Philadelphia, Pennsylvania, USA.

Qin Li and Yingxin Li contributed equally to this work.

* Correspondence should be addressed to**:** jyan1@mdanderson.org (Jiusheng Yan).

Supplementary Information

Table S1. Boltzmann fit parameters of voltage-dependent BK channel activation.

	0 $[Ca^{2+}]_i$			1.5 µM $[Ca^{2+}]_i$			7.5 µM $[Ca^{2+}]_i$			90 µM $[Ca^{2+}]_i$			1 mM $[Ca^{2+}]_i$			$\Delta V_{1/2\ (-/+Ca)}$ (mV)			
	$V_{1/2}$ (mV)	z (e)	*n*	$V_{1/2}$	z	*n*	$V_{1/2}$	z	n	$V_{1/2}$	z	*n*	$V_{1/2}$	z	*n*	1.5 µM	7.5 µM	90 µM	1 mM
WT	167.5 ± 2.4	1.26 ± 0.07	8	98.2 ± 4.0	1.46 ± 0.13	4	18.7 ± 2.8	1.54 ± 0.06	6	-31.6 ± 2.8	1.50 ± 0.09	7	-68.7 ± 2.2	1.63 ± 0.11	4	69.3 ± 4.7	148.8 ± 3.7	199.1 ± 3.7	236.3 ± 3.3
N449A	193.2 ± 5.3	1.10 ± 0.10	6	126.5 ± 2.6	1.20 ± 0.04	4	53.1 ± 2.0	1.46 ± 0.10	6	-10.3 ± 3.9	1.42 ± 0.09	7	-39.8 ± 2.9	1.25 ± 0.06	4	66.7 ± 5.9	140.1 ± 5.7	203.5 ± 6.6	233.0 ± 6.0
E955Q	127.2 ± 1.4	1.18 ± 0.09	4	106.0 ± 8.0	1.33 ± 0.08	4	62.9 ± 5.2	1.30 ± 0.06	6	21.3 ± 2.6	1.21 ± 0.05	6	-25.3 ± 4.1	1.43 ± 0.10	3	21.2 ± 8.1	64.3 ± 5.4	105.9 ± 2.9	152.5 ± 4.3
E956Q	160.4 ± 4.2	1.16 ± 0.06	4				70.6 ± 6.3	1.06 ± 0.10	4	-11.6 ± 6.2	1.66 ± 0.07	5					89.8 ± 7.5	172.0 ± 7.5	
E955Q/E956Q	128.4 ± 3.8	1.23 ± 0.07	3				92.8 ± 4.6	1.08 ± 0.13	3	23.2 ± 1.1	1.31 ± 0.04	5					35.6 ± 6.0	105.2 ± 4.0	
5D5N	162.8 ± 5.0	1.37 ± 0.09	7				113.5 ± 4.6	1.22 ± 0.06	5	53.3 ± 3.1	1.29 ± 0.11	6					49.3 ± 6.7	107.5 ± 5.8	
5D5N/E955Q	114.5 ± 3.5	1.39 ± 0.10	6				64.5 ± 1.5	1.23 ± 0.08	4	35.1 ± 4.2	1.31 ± 0.10	4					50.0 ± 3.8	79.3 ± 5.5	
D362A/D367A	152.9 ± 2.5	1.06 ± 0.07	6				87.3 ± 3.0	1.34 ± 0.11	3	47.9 ± 1.6	1.22 ± 0.07	5					65.6 ± 3.9	105.0 ± 2.9	
D362A/D367A/E955Q	113.6 ± 3.0	1.38 ± 0.09	5				77.4 ± 3.8	1.31 ± 0.04	8	64.6 ± 2.7	1.28 ± 0.03	5					36.2 ± 4.8	49.0 ± 4.0	
R786A	151.8 ± 4.8	1.48 ± 0.05	5				40.3 ± 6.1	1.56 ± 0.10	5	7.1 ± 3.5	1.27 ± 0.09	5					111.5 ± 7.8	144.7 ± 5.9	
R790A	158.9 ± 4.2	1.30 ± 0.09	6				69.6 ± 6.6	1.30 ± 0.10	4	7.3 ± 4.3	1.42 ± 0.17	3					89.3 ± 7.8	151.6 ± 6.0	
R786A/R790A	142.3 ± 2.9	1.52 ± 0.09	5	96.5 ± 3.4	1.67 ± 0.08	6	52.0 ± 1.6	1.48 ± 0.10	6	9.7 ± 3.3	1.18 ± 0.09	4				45.8 ± 4.5	90.3 ± 3.3	132.6 ± 4.4	
R786A/R790A/E955Q	129.4 ± 3.8	1.49 ± 0.10	6	77.0 ± 4.1	1.70 ± 0.11	6	17.5 ± 3.9	1.34 ± 0.08	6	-19.0 ± 3.9	1.36 ± 0.07	6				52.4 ± 5.6	111.9 ± 5.4	148.4 ± 5.4	

n values are the number of recorded excised inside-out patches from different HEK-293 cells.

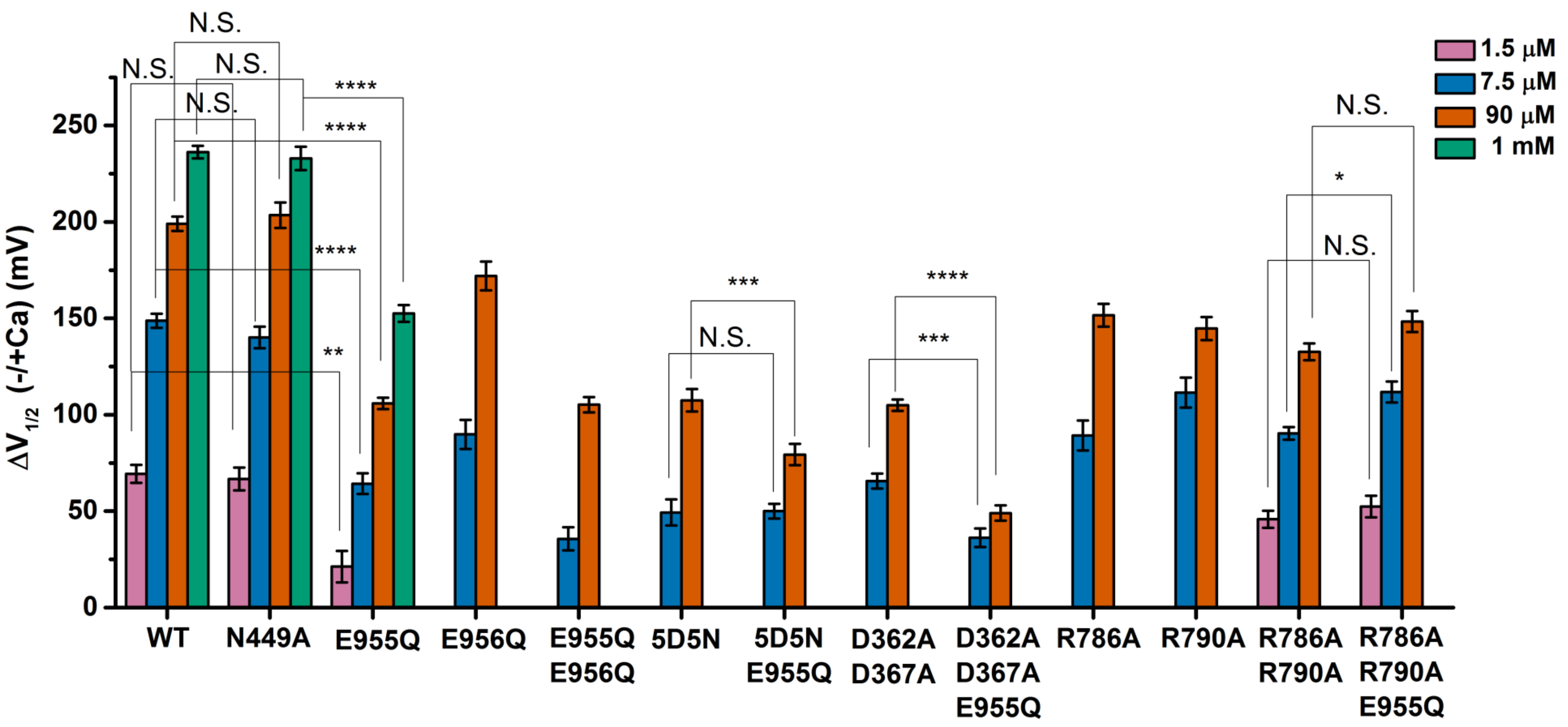

Figure S1. Ca^{2+}-induced shifts in $V_{1/2}$ in WT and different mutant channels. Statistical differences were evaluated with a *t* test indicated with N.S. for p >0.05 (not significant), ** for p <0.01, *** for p <0.001, and **** for p <0.0001.
